# Thermal Performance of Mortars Based on Different Binders and Containing a Novel Sustainable Phase Change Material (PCM)

**DOI:** 10.3390/ma13092055

**Published:** 2020-04-28

**Authors:** Antonella Sarcinella, José Luìs Barroso De Aguiar, Mariateresa Lettieri, Sandra Cunha, Mariaenrica Frigione

**Affiliations:** 1 Innovation Engineering Department, University of Salento, Prov. le Lecce-Monteroni, 73100 Lecce, Italy; antonella.sarcinella@unisalento.it; 2 Civil Engineering Department, University of Minho, Campus de Azurém, 4800-058 Guimarães, Portugal; aguiar@civil.uminho.pt; 3 CNR—SPIN, via Giovanni Paolo II 132, 84084 Fisciano (Salerno), Italy; mariateresa.lettieri@cnr.it; 4 Lusophone University of Humanities and Technologies, Campo Grande 376, 1749-024 Lisboa, Portugal; sandracunha86@gmail.com

**Keywords:** phase change material (PCM), thermal energy storage (TES), sustainable materials for buildings, thermal properties, mortars, hydraulic lime, cement

## Abstract

Increasing concerns about climate change and global warming bring about technical steps for the development of several energy-efficient technologies. Since the building sector is one of the largest energy users for cooling and heating necessities, the incorporation of a proper energy-efficient material into the building envelopes could be an interesting solution for saving energy. Phase change material (PCM)-based thermal energy storage (TES) seems suitable to provide efficient energy redistribution. This is possible because the PCM is able to store and release its latent heat during the phase change processes that occurs according to the environmental temperature. The purpose of this paper was the characterization of the thermal properties of a composite PCM (i.e., Lecce stone/poly-ethylene glycol, previously developed) incorporated into mortar compositions based on different binders (i.e., hydraulic lime and cement). The study was carried out using an experimental set up through which it was possible to simulate the different seasons of the years. It was observed that the addition of PCM in mortars leads to a decrease of the maximum temperatures and increase of the minimum temperatures. Furthermore, the results shown a reduction of the heating and cooling needs, thus confirming the capability of this material to save energy.

## 1. Introduction

In the current world scenario, environmental issues as well as climate change represent a real problem that concerns all humanity. This awareness has led international policy to incentivize research pushing it in the development of new renewable energy solutions. On the other hand, the scientific community is focused on it and trying to make important steps forward to limit the increase in energy consumption. In the last few decades, it has been shown that the building sector contributes significantly to the increase the electric energy demands [1]. Major components of energy consumption in the building sector are in heating, cooling, air conditioning and ventilation systems for comfort demands [2]. For this reason, buildings should be designed to ensure the thermal comfort of the occupants, with minimum auxiliary energy for heating and cooling equipment [3].

Another problem associated with energy consumption is due to old and ancient constructions characterized by a lack of building envelope insulation; in the case of historical buildings, moreover, the installation of modern devices for heating and cooling necessities is limited. For this reason, it becomes essential to find solutions able to improve the energy efficiency of buildings preserving, at the same time, the environment [4].

A potential tool for energy conservation, able to store the excess energy and to release it, filling the gap between the energy supply and demand, is a system called thermal energy storage (TES).

This latter system, using the principle of latent heat thermal energy storage (LHTES), combined with a proper phase change material (PCM) is a promising technology to save energy, improving building efficiency [5]. As the temperature increases, the PCM has the capability to change its phase from solid to liquid. The reaction is endothermic and the PCM absorbs heat; when the temperature decreases, the material changes its phase from liquid to solid. At this point, the process is exothermic and the PCM desorbs heat. Thus, the addition of a PCM in building service equipment is a way to enhance energy storage capacity. Consequently, building energy performance could be optimized and also indoor thermal comfort can be improved [6]. The incorporation of a PCM in construction materials (passive building system) has proven to be the most interesting approach [7].

Thereby, wallboards, floors, roof, mortar or concrete and other parts are integrated with PCMs in order to improve the thermal performance of the building [8,9,10,11,12]. The most common solution for implementing PCMs in buildings is the installation of PCM into the interior side of the building envelope. Thus, the use of suitable PCMs in the interiors of the construction allows absorbing and releasing heat in any room during a large part of the day. Several experimental investigations have shown how this strategy positively affects indoor climate and energy use [6,7,13,14]. On the other hand, among all these possible applications, the incorporation of phase-change materials in mortars employed in the interiors of buildings appears the most attractive solution in an attempt to minimize the massive energetic consumption related to building conditioning. Such an approach allows the regulation of the temperature inside buildings through latent heat energy storage, using only solar energy as a resource, thus, reducing the need for heating/cooling equipment [15,16]. Incorporating PCMs in mortar and concrete can be an efficient method due to the large heat exchange area surfaces; in addition, the final functional material can be adapted in a wide variety of shapes and sizes. Being mortar and concrete widely used as construction materials, such PCM composites can be employed in any practical application. Moreover, quality control can be easily achieved in the materials produced [17].

Among the available methods to incorporate an effective PCM into a building material, such as mortar or concrete, the “form stable” is among the simpler and more efficient technologies [3,17,18,19]. In this method, a porous matrix of inorganic (such as silica-based material, perlite, diatomite, clay, etc.) can act as the inert support that will contain the true PCM [20,21]. A form-stable PCM composite can then be obtained by immersing the matrix in the liquid PCM, employing a vacuum system to force the impregnation. After the impregnation process, the porous matrix is able to retain the optimal percentage of PCM, with no leakage [22,23]. The production of form-stable PCMs involves very cheap and simple equipment. Several studies have been recently published on the use of construction materials incorporating a form-stable PCM composite [21,24,25,26].

In this work, this route (i.e., the form-stable method) was followed, employing as support matrix small pieces of Lecce stone (LS) supplied by a local quarry as a waste product and selecting low toxic and low flammable PEG (poly(ethylene glycol)) as real PCM. The use of waste stone as support for a PCM able to improve the thermal efficiency of the buildings, and to reduce the consumption of petroleum-derived energy, would represent a double advantage for the environment. PEG was chosen because of its favorable properties, such as suitable phase change temperatures and large phase change enthalpy, elevated long-term thermal/chemical stability, low toxicity and resistance to corrosion, and limited volume change during solid–liquid phase change [27,28]. Furthermore, the range of melting/crystallization temperatures of the selected grade of PEG (i.e., 1000) were considered particularly suitable for a PCM included in mortars to be used in Mediterranean warm countries.

The produced form-stable PCM system was then used as an aggregate for the production of some mortar formulations based on different binders. In our previous works [29,30], the production and the optimization process to achieve a suitable form-stable composite PCM have been described, as well as its chemical, physical and thermal characterization. The influence of this PCM composite system included in different mortar compositions on their mechanical properties was also investigated [30,31].

Starting from the previous research, taking into account the results obtained, the main aim of the study described in this paper was the analysis of the thermal performance of some of the produced mortars exposed to temperatures currently recorded in the South of Italy. To this purpose, an experimental setup was implemented: a small-scale test cell, on which the produced mortars (with and without PCM) were poured, was submitted to a preset temperature program able to simulate the different seasons of the year. The obtained thermal performance for each binder mix, unmodified or containing the innovative PCM, was recorded and analyzed.

## 2. Materials and Methods

### 2.1. Production of the Form-Stable Phase Change Material (PCM)

Different mortar compositions to be used indoors were developed in previous studies [30,31].

Lecce stone (LS), a biocalcarenite typical of the Salento area (South Italy) with a high open porosity [32], was selected as support matrix to contain the PCM. LS was chosen also because it is widely available as a waste product from extraction and production of stone components. Lecce stone was reduced in small pieces and sieved up to a granulometry ranging between 1.6 and 2.0 mm, to be used as aggregate in the mortar formulations. The PCM was based on poly (ethylene glycol), supplied in solid form (Sigma–Aldrich company, Germany) with the trade name PEG 1000.

According to the data sheet, the density of PEG 1000 at 20 °C is 1.2 g/cm^3^. PEG 1000 displays a mix of favorable properties (cheapness, low toxicity, low flammability) along with suitable melting and crystallization range of temperatures. In order to be used in building applications, in fact, a PCM must display an appropriate range of phase transition temperatures. Since PEG shows melting and crystallization temperatures of 43 °C and 23 °C, respectively, this polymer has been considered favorable to be used as a Phase Change Material included in mortars to be employed in buildings located in warm regions, for instance in the Mediterranean area [27,28].

To produce the PCM composite system, namely LS/PEG, a simple vacuum impregnation process was employed, according to the form-stable principle. As reported in our previous work [30], a proper quantity of LS granules, that constituted the support for the PCM, was positioned in a flask linked to a vacuum pump (at a vacuum pressure of 0.1 MPa). This flask was placed on a magnetic stirrer, kept at 60 °C, containing also a magnetic stir bar. The PEG (originally in solid form), constituting the true phase change component, was heated at 80 °C and then added to the flask where the LS granules were placed. At that point, air was allowed to enter in the flask, forcing the penetration process of the liquid PEG into the support, i.e., in the pores of LS. This process was continued for 60 min. The results achieved with this method, reported in [30], have shown that the maximum percentage of PEG absorbed in LS is 23% by weight. It has been demonstrated that in this form stable PCM displays appropriate LHTES properties to be used in indoor mortars employed in buildings located in warm (for instance, Mediterranean) regions [27].

### 2.2. Compositions of Mortar Formulations

In our previous papers, different mortar formulations based on aerial lime, hydraulic lime, gypsum and cement, containing the composite LS/PEG have been produced and investigated [30,31], with the aim of identifying, for each binder, the most convenient composition able to produce mortars with adequate mechanical properties, in terms of both flexural and compressive modes. The mix designs based on hydraulic lime and cement mortars were then identified as those best performing. These latter were, therefore, employed to assess the true capability of LS/PEG to improve the thermal regulation of a building, decreasing the indoor maximum temperature and increasing the indoor minimum temperature. The selected binders, moreover, can be used in different applications: for historical constructions the hydraulic lime, the cement-based mortars in modern buildings and for any other common application [30,31].

A natural hydraulic lime (NHL), with density of 2700 kg/m^3^ supplied by CIMPOR (Lisbon, PT) was selected. A CEM I 42.5 R cement, with a density of 3030 kg/m^3^, supplied by SECIL (Lisbon, PT), was employed in the present work. The inert support for the PEG-based PCM is the stone (LS). Additional information of these mortars and its raw materials can be found in [31].

A superplasticizer (SP) was added to both mortar compositions, in order to reduce the amount of water required for the mixing. The SP was a polyacrylate (MasterGlenium SKY 627, supplied by the BASF company), with density of 1050 kg/m^3^. In Table 1, the composition of all the mortars realized and analyzed, produced according to the European Standard EN 998-1 [33], are reported.

Four compositions were developed: two of them were produced by adding the composite LS/PEG to the binders (i.e., HL_800__LS/PEG and C_800__LS/PEG), in order to evaluate the effect of the presence the PCM on the thermal properties of different mortars. For comparison purposes, two control formulations were prepared by introducing only LS as aggregate (i.e., HL_800__LS and C_800__LS). As for the mortar compositions containing the composite PCM (i.e., LS/PEG), the amount of this latter is different in the two produced mortars (based on hydraulic lime and cement, respectively) since the amount of each component in the mortars was determined according to Equation (1), where each component is related to its density:

(1) $$\frac{Binder\;\left(\mathrm{kg}\right)}{\rho_{b}}+\frac{Aggregates\;\left(\mathrm{kg}\right)}{\rho_{a}}+\frac{Superplasticizer\;\left(\mathrm{kg}\right)}{\rho_{s}}+\frac{Water\;\left(\mathrm{kg}\right)}{\rho_{w}}=1\;m^{3}$$

where $\rho_{b}$, $\rho_{a}$, $\rho_{s}$ and $\rho_{w}$ are the densities of the binder, the aggregates, the superplasticizer and the water, respectively.

The indication “water saturation” in Table 1 accounts for the water used to saturate the LS aggregates, possessing a high porosity, to prevent them from absorbing the water necessary for the mortar’s manufacture. This additional water was not required when LS/PEG composite was added to mortars, since PEG was able to saturate the pores of Lecce stone [30].

### 2.3. Methods

The morphology and microstructure of the developed mortars were analyzed using a scanning electron microscope (SEM, Carl Zeiss Auriga40 Crossbeam instrument, Jena, Germany). The investigations were performed under vacuum on samples without metallization, using a beam accelerating voltage of 20 kV and the secondary electron detector. The SEM images were further analyzed using the Scanning Probe Image Processor (SPIP) software package (Image Metrology A/S, Kgs. Lyngby, Denmark), version 6.2.6. Three images at magnification of 2500× were analyzed for each material and the results were averaged. The “Particle and Pore analysis” function was used to detect the pores and to measure their size. The threshold segmentation method was applied to quantify the total pores percentage; the watershed method was used to evaluate the distribution of the pores on the basis of their dimension.

The thermal behavior of the produced mortars was tested in a climatic chamber, setting a temperature program representative of each season of the year, in order to study the thermal behavior of the developed mortars in summer, spring, autumn and winter. The temperature program was fixed based on climatic data collected at the weather station installed in the Salento region, South Italy.

**Figure 1:**
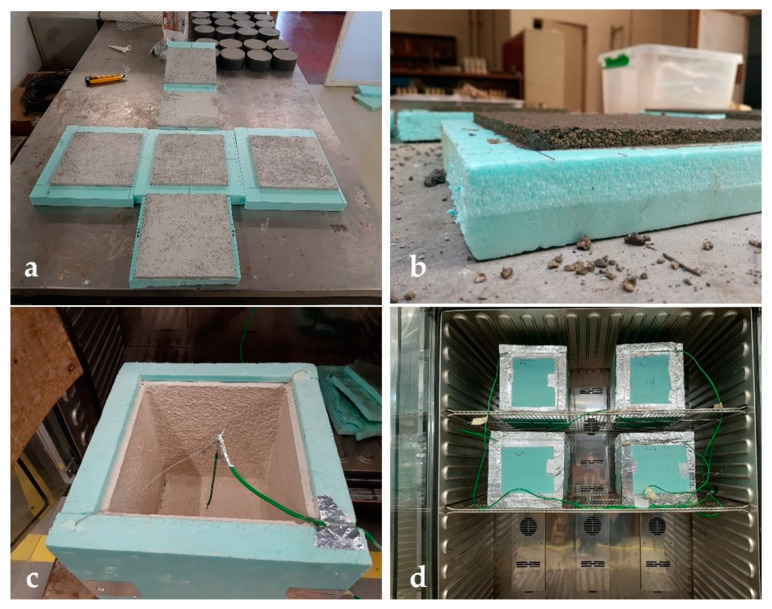
Set up to investigate the thermal performance of the different mortars: (**a**) six faces to build a small-scale test cell; (**b**) mortar layer of 1 cm; (**c**) small-scale test cell with a thermocouple in the center; (**d**) small-scale test cells placed inside a climatic chamber with temperature control.

For each composition, a small-scale test cell (Figure 1a) was constructed with an insulating material (polystyrene) 3-cm thick, coated in the internal side with a mortar layer of 1 cm (Figure 1b). Each cell was a cube, with a side equal to 200 mm. A thermocouple was placed in the center of each small-scale cell, as illustrated in Figure 1c. Each small-scale test cell was placed inside a climatic chamber, equipped with thermocouples for the temperature control (Figure 1d). Each thermocouple used during the tests was connected to a data acquisition system of high sensibility (AGILENT 34970A), measuring every minute the temperature of the climatic chamber and that inside the small-scale test cells; the measured temperatures were then recorded by software (BenchLinkDataLogger3). Type K thermocouples were used. The described set-up is able to supply important and reliable information on the thermal behavior of the mortars, as previously demonstrated [34,35].

## 3. Results and Discussion

### 3.1. Microstructure 

**Figure 2:**
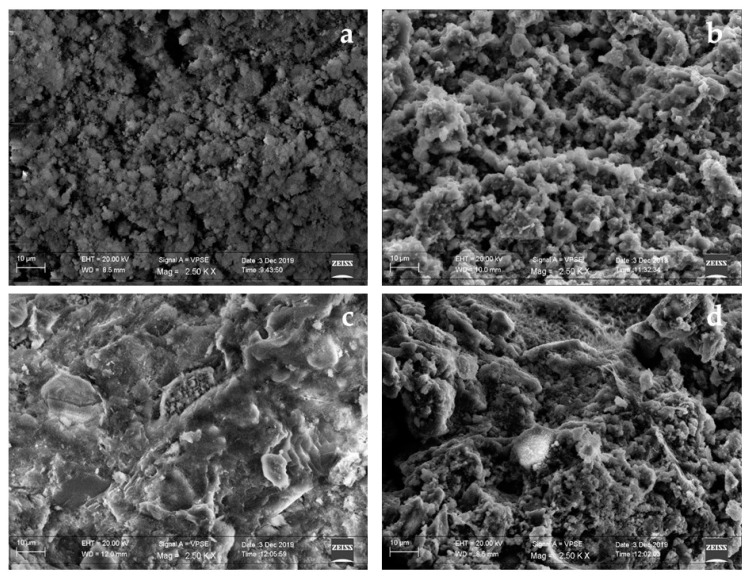
Scanning electron microscope (SEM) images of (**a**) hydraulic lime-based mortar without phase change material (PCM) (HL_800__LS (Lecce stone)); (**b**) hydraulic lime-based mortar with PCM (HL_800__LS/PEG (poly(ethylene glycol))); (**c**) cement-based mortar without PCM (C_800__LS) and (**d**) cement-based mortar with PCM (C_800__LS/PEG).

The scanning electron microscope observations were performed to evaluate possible incompatibilities between the different phases present in the mortars. Figure 2 shows the microstructure of the hydraulic lime and cement based mortars with incorporation of the composite system, i.e., PEG-based PCM, compared with the reference mortars without PCM.

**Figure 3:**
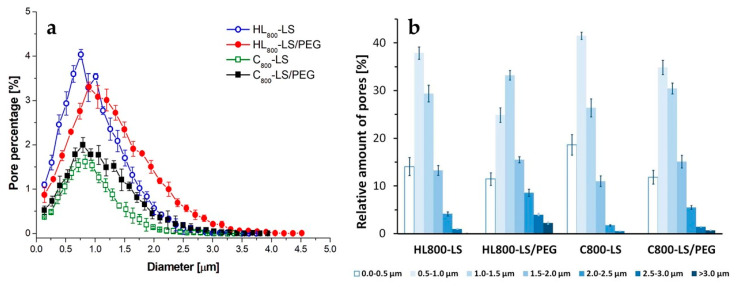
Porosimetric features by image analysis of the SEM micrographs in Figure 2: (**a**) percentage of pores as a function of their diameter; (**b**) number of pores per classes of pore-size.

The absence of voids and cracks in the microstructure of the developed mortars, irrespective to their composition, suggests a good connection between the different components (LS or LS/PEG and each binder). Differences in the pore size and pore distribution were found when comparing the mortars with and without PCM, as can be observed from the results in Figure 3. A slight increase in the total pores was measured when the PCM was added to each mortar (Table 2). Taking into account that the total pores account for the porosity of the material, the mortars based on hydraulic lime were generally more porous than the cement-based ones, as expected. The most frequent pore size was similar for all the analyzed materials; it ranged between 0.761 and 0.894 µm (Figure 3a and Table 2). In addition, the mortars containing the PCM exhibited a higher percentage of larger pores, irrespective of the kind of binder. The fraction of pores larger than 2 µm was approximately 15% in HL_800__LS/PEG; it decreased by half in C_800__LS/PEG, while it was negligible in the most compact mortar (i.e., C_800__LS).

In general, the presence of both higher porosity and larger pore size can be explained in terms of a higher water content of the mortar’s formulation. As reported in Table 1, the mortar compositions containing LS/PEG were prepared using a higher amount of water. Thus, both hydraulic lime and cement-based mortars with PCM exhibited increased porosity and pores larger in dimension than the corresponding controls due to the higher water content employed. The observed porosimetric features can also account for the changes in mechanical properties; in fact, the addition of the PCM brought about a certain decrease in mechanical strength if compared to the mortars not containing the LS/PEG composite.

### 3.2. Thermal Behavior

**Figure 4:**
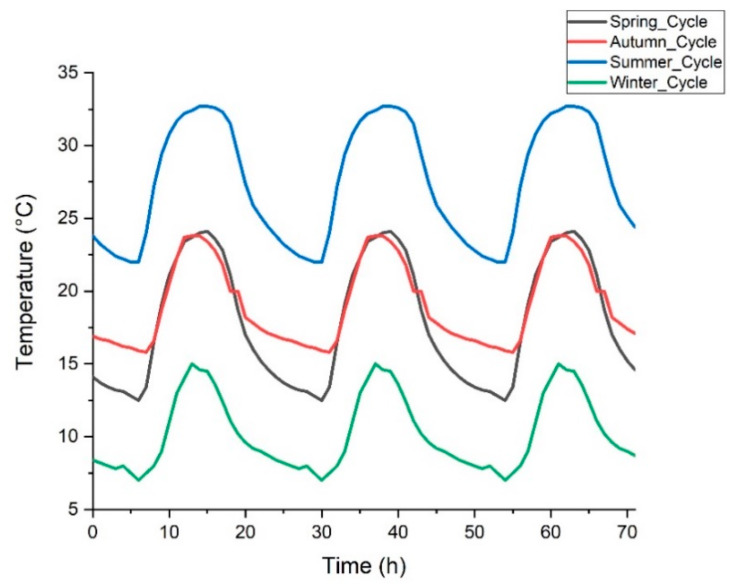
Temperature programs used to simulate the different season of the year.

Since the presence of a PCM is expected to positively influence the internal temperature of a room where it is applied [3,11,34], thermal tests were conducted with the aim of evaluating the thermal behavior of mortars with incorporation of PCM. During the tests, all seasons of the year were evaluated, taking as reference the climate recorded in Salento region (South Italy). Poly-ethylene glycol 1000 was, in fact, selected as PCM since its range of melting/crystallization temperatures is suitable for the intended purpose. The summer conditions were simulated employing a temperature range from 22 °C to 32 °C. For the spring climate, the analyzed temperature ranged between 12 °C and 24 °C. In the autumn, the temperatures ranged between 16 °C and 24 °C. To simulate the winter, a temperature interval between 8 °C and 15 °C was selected. Figure 4 shows the characteristic temperatures used to simulate a typical summer, spring, autumn and winter season in Salento region. Each season was simulated with three cycles, each one with a duration of 24 h.

During these tests, the PEG-based PCM reached the phase transition (between 27 and 30 °C during the heating process and between 10 and 13 °C during the cooling process) storing and releasing energy from the environment, respectively, regardless of the kind of binder. These temperatures are reported to be, in theory, favorable to obtain a PCM-based mortar to be employed as thermal energy storage system included in the exterior and/or in indoor walls of buildings located in warm regions [30,31]. In the case of winter season, it was not possible to evaluate the PCM effect since its melting point was hardly achieved [31], therefore, these data were not reported.

**Figure 5:**
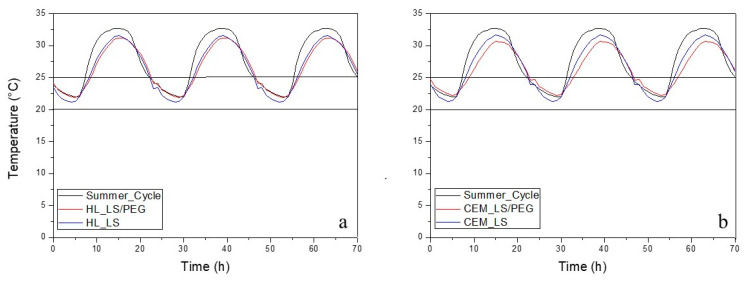
Thermal behavior in summer of the developed mortars: (**a**) hydraulic lime-based mortar and (**b**) cement-based mortar. The thermal comfort zone is between 20–25 °C.

**Figure 6:**
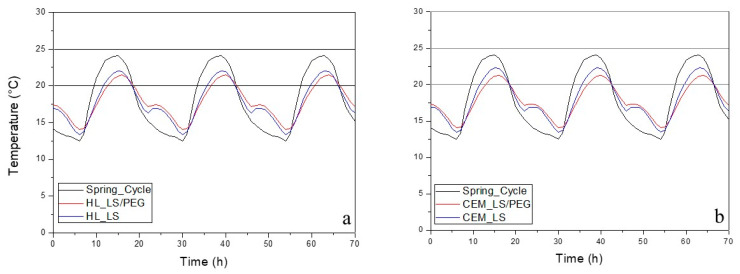
Thermal behavior in spring of developed mortars: (**a**) hydraulic lime-based mortar and (**b**) cement-based mortar. The thermal comfort zone is between 20–25 °C.

**Figure 7:**
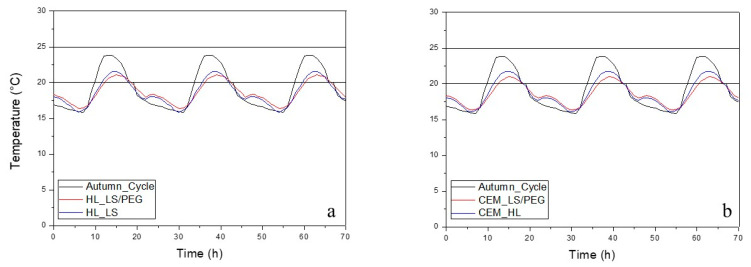
Thermal behavior in autumn of developed mortars: (**a**) hydraulic lime-based mortar and (**b**) cement-based mortar. The thermal comfort zone is between 20–25 °C.

In Figure 5, Figure 6 and Figure 7 report the behavior of the developed mortars, i.e., lime-based and cement-based mortars, with and without the novel PCM, in spring, summer and autumn climatic conditions. In each graph, the fluctuations of the typical temperatures for each season, set in the climate chamber where the test cells were located, are also reported. Figure 5 shows the thermal behavior of the developed mortars during summer. It can be observed that temperatures above 25 °C are registered but not below 20 °C. Thus, it was concluded that during the summer there are cooling needs. Figure 6 shows the thermal behavior of the mortars during the spring. Cooling is not necessary since, for the mortars containing the PCM, the maximum temperature is lower than 25 °C. Finally, Figure 7 shows the thermal behavior during the autumn: even in the presence of the PCM-based mortar, slight heating is needed, since the minimum temperatures were lower than 20 °C. In all the performed tests it was observed that when the temperature inside the test scale-cell achieves the range between 20 °C and 25 °C, a slight PCM phase change occurs: the thermal behavior of the PCM-based mortars evolves in a different way compared to the temperature program used for the simulation of the season. It was verified that the temperatures inside the test cells did not reach such extreme temperatures if compared to the temperature program. Furthermore, the temperature fluctuations of the mortar compositions containing the PCM are always narrow if compared to those measured on the mortar compositions without PCM, conforming the efficient behavior of the LS/PEG composite as phase change material for different mortars. 

As for the summer climatic condition (Figure 5), it was observed that in the heating step, i.e., when the temperature exceeds 25 °C, the PCM-based mortars showed a slower heating and a lower maximum temperature, this behavior being more marked in the case of the cement-based mortar. When the temperature lies near the indoor thermal comfort zone (20–25 °C), the cells exhibit similar temperature values. The effect of heat storage/release is only detected when the temperature diverges from the thermal comfort zone. As for the cement-based mortar, it was observed that the higher temperature of C_800__LS/PEG is lower than 2 °C compared to the temperature program, while the temperature of C_800__LS is lower than 1 °C if compared to the temperature program. Thus, starting from a decrease of the maximum temperature greater than 3% in the cooling stage achieved for the cement mortar, a decrease of the maximum temperature greater than 6% is achieved when the PCM is added to the same mortar formulation. Passing to analyzing the hydraulic lime-based mortar, on the other hand, the differences between the mortar formulation with and without PCM were negligible. During the same tests, it was also observed a lag time of the minimum temperatures of 60 min during the heating stage, while no lag time was recorded during cooling stage (Table 3). During the summer the greater part of residential buildings electricity consumption is used for cooling needs, thus a certain shift to off-peak periods of this consumption can represents an economical advantage.

According to Figure 6, in the spring climate, the maximum temperature did not exceed 25 °C, meaning that the indoor environment does not require any cooling. On the other hand, the minimum temperatures are lower than 20 °C and, consequently, there are heating necessities. As can be seen in the graphs shown in Figure 6, the lowest temperature, recorded inside the climatic chamber and representative of the spring cycle, diverges from the temperature recorded inside the test cells. In general, there are not significant differences between the different binders (i.e., hydraulic lime and cement), since for both of them the incorporation of PCM leads to an increase in the minimum temperature of 11%. In particular, it was observed that the lower temperature of C_800__LS/PEG is greater than 2 °C when compared to the temperature program, while the temperature of C_800__LS is greater than 1 °C with respect to the temperature program. The same can be said for the hydraulic lime-based mortar. However, in this tested season, the effect of the energy storage/release of the PCM is found also in the thermal comfort zone (20–25 °C). The incorporation of PCM into mortars leads to a decrease of 12% in the maximum temperature. For the cement-based mortar as well as for the hydraulic lime-based one containing the PCM, the higher temperature was found to be lower than 3 °C compared to the temperature program, while the temperatures of the same mortar formulations without the PCM are lower than 2 °C if compared to the temperature program.

**Figure 8:**
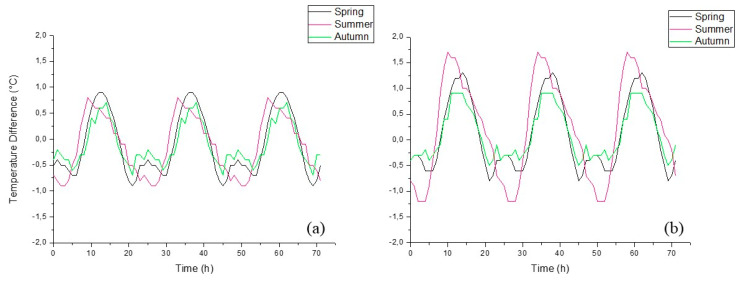
Thermal gradient between unmodified and PCM-based mortars: (**a**) hydraulic lime-based mortars and (**b**) cement-based mortars.

These results demonstrated that the PCM produced positively influences to a similar extent both high- and low-temperature external conditions. For the spring season, since there are not substantial differences between the different mortar compositions in terms of thermal regulation, the only difference can be highlighted by the thermal gradient, as can be seen in Figure 8, where the better thermal behavior of the cement-based mortar appears remarkable.

Table 4 shows the lag time of the maximum and minimum temperatures verified in the different mortars with and without PCM incorporation. It was observed that there is a lag time of the maximum temperature of 60 min in the cooling stage, no lag time of the minimum temperature is observed in the heating stage.

Figure 7 shows the thermal performance of the different mortars in an autumn climate. It can be observed that the lowest temperatures are outside the comfort temperature zone since they are lower than 20 °C. This observation suggests that it could be necessary to use heating equipment, leading to higher energetic consumption in buildings. Starting from the mortar compositions containing the PCM (i.e., C_800__LS/PEG and HL_800__LS/PEG), it was possible to obtain an increase in the lowest temperature of 4%. This means that the difference between the temperature program, representative of the autumn season, and the test cells containing the mortars with the PCM is about 1 °C. On the other hand, the increase in temperature for the mortar compositions without the PCM is irrelevant if compared to the temperature program. However, a decrease in the maximum temperature of 12% was also measured for both mortar formulations containing the PCM. For the cement-based mortar as well as for the hydraulic lime-based one containing the PCM, the higher temperature was found to be lower than 3 °C when compared to the temperature program, while the temperature of the same formulations without the PCM is lower than 2 °C with respect to the temperature program. Thus, it was concluded that the PCM is able to more greatly influence a cooling stage if compared to a heating one.

According to the results reported in Table 5, the lag time of the maximum temperature in the cooling stage is 60 min; no lag time of the minimum temperature was recorded in the heating stage.

The analysis of the experimental temperature curves described does not provide enough information about the energy performance of the different mortars subjected to heating and cooling cycles. Therefore, it is necessary to measure the temperature differences within each cell relative to the PCM-based mortars with respect to the reference ones (0% PCM). The thermal gradient was determined for each hour of the thermal tests and calculated by Equation (2).

ΔT = T_ref_ − T_PCM_ (2)

where T_ref_ is the temperature of reference mortars (0% PCM) (°C); T_PCM_ is the temperature inside the cell with PCM-based mortars (°C).

Figure 8 shows the variation of the thermal gradient for each mortar composition in each season. As the temperature cycle runs, the thermal gradient increases as a result of the cyclic heat storage process. Hence, the gradient decreases until it reaches the point where the cells have the same temperature (ΔT = 0). The cement-based mortars exhibit better thermal regulation with the greater difference in the temperatures observed in all the season tested. On the other hand, hydraulic lime-based mortar exhibit a smaller thermal gradient and, as a consequence, lower thermal performance.

Based on the temperature curves (Figure 5, Figure 6 and Figure 7), the reduction of energy consumption during the cooling and heating cycles was also quantified, with the aim of evaluating the energy saving actually achieved with the use of the novel PCM. The amount of energy required to maintain the temperature of the cell inside the temperature comfort range during one day was then calculated for each season. In this way, it was possible to have an estimation of the cooling and heating needs to maintain the interior temperature of a building within the comfort temperature range, i.e., between 20 °C and 25 °C. Table 6 presents the cooling and heating needs for the different mortars in the different seasons. 

It was observed that the incorporation of the novel PCM, through the aggregates LS/PEG, into mortars caused a slight decrease for the cooling needs in summer season. In this season, cement-based mortar shows a better thermal behavior, since lower cooling needs are required using this mortar composition.

As for the spring climate, a decrease was calculated in the heating needs for both the formulations, hydraulic lime, and cement-based mortar, containing the PCM. The differences between the two different binders appear to be insignificant. In the autumn conditions, a very small decrease was calculated in the heating needs due to the incorporation in the mortars of the LS/PEG composite. Once again, the differences between HL_800__LS/PEG and C_800__LS/PEG are not significant. From the performed thermal tests was, then, possible to confirm that the PCM incorporation in both mortars formulations reduces, even if to a low extent, the cooling and heating needs of a building located in Salento region depending on the simulated season.

In Table 7 the values of energy saving that could be achieved using both mortar formulations containing the PCM composite are reported. This latter was determined according to the difference of the energy required for cooling and heating needs, starting from the results reported in Table 6 and calculated by Equation (3):

**ΔNE = NE_0PCM_ − NE_PCM_** (3)

where NE is the reduction of energy needs (J/m^3^); NE_0PCM_ is the energy required for mortar without PCM (J/m^3^); NE_PCM_ is the energy required for mortar with PCM (J/m^3^).

In summer, when only refreshment is required, the cement-based mortar displays a better thermal behavior, confirmed by the much greater amount of the energy saving. In spring, on the other hand, the energy saving for the hydraulic lime-based mortar is almost 20% greater than that calculated for the cement-based mortar. The advantage of hydraulic lime-based mortar is even more appreciable during the autumn season, with a difference of 40% in energy saving compared to cement-based mortar. This behavior could be ascribed to the greater PEG content and porosity of the hydraulic lime mortar formulation (Figure 1 and Figure 2). Thus, it can be concluded that the presence of a greater amount of macro pores improves the temperature regulation effect.

## 4. Conclusions

In this work, the influence of a composite phase change material system included into two mortar compositions, based on cement and on hydraulic lime, on thermal properties of the same mortars was evaluated. The PCM represents a novel sustainable composite system composed of eco-friendly PEG included, through the “form-stable method”, in waste natural stone flasks, i.e., Lecce stone. This system, prepared with a low-cost equipment and low-cost raw materials, is characterized by other favorable properties, such as low flammability and low toxicity that constitute advantageous features for applications in the building sector. 

The microscopic analysis of the microstructure of each mortar revealed a good connection between the different components of the mortars, with no voids or cracks in a homogeneous structure. From the same analysis it was also possible to observe that the incorporation of LS/PEG led to an increase in the porosity in the tested binders in comparison with the reference unmodified mortars. This observation was explained by the higher amount of water required by the mortars containing the PCM, with the hydraulic lime-based mortar containing LS/PEG showing the greatest porosity.

The results obtained in the thermal tests, performed with a small-scale test cells, simulating the temperatures typically experienced in the Salento region (South Italy) in the four seasons, have proven that the incorporation of the PCM under analysis in the mortars leads to changes in their thermal properties. In particular, during the summer season, a decrease in the cooling needs was measured, while during spring and autumn, a decrease in the heating needs can be achieved upon the addition of the PCM in both mortars. The best thermal performance was achieved in the summer season by the cement-based mortar containing the LS/PEG, testified by the highest thermal gradient, the maximum temperature reduction, the minimum temperature increase and time delay. In spring and autumn, the best thermal performance was displayed by the hydraulic lime-based mortar containing the LS/PEG.

## Figures and Tables

**Table 1:** Mortar compositions (reported as kg/m^3^ of produced mortar).

System	Binder/Content	Aggregates		SP	Water Saturation	Water	Water/Binder
		Lecce Stone (LS) Content	Poly Ethylene Glycol (PEG) Content				
HL_800_LS	Hydraulic Lime/800	1092	0	15	275	320	0.40
HL_800_LS/PEG	Hydraulic Lime/800	1729	398	15	0	375	0.47
C_800_LS	Cement/800	1070	0	15	269	296	0.37
C_800_LS/PEG	Cement/800	1347	310	15	0	360	0.45

**Table 2:** Total pores as a percentage and the most frequent pore size obtained by image analysis of SEM micrographs taken on the investigated mortars.

System	Total Pores (%)	Most Frequent Pore Size (μm)
HL_800__LS	33.57 ± 3.53	0.761 ± 0.062
HL_800__LS/PEG	36.57 ± 3.18	0.894 ± 0.075
C_800__LS	14.57 ± 1.35	0.832 ± 0.057
C_800__LS/PEG	19.66 ± 3.24	0.794 ± 0.076

**Table 3:** Lag time between the maximum and minimum temperatures in summer climate.

Summer	Lag Time (min)	
	Cooling Stage	Heating Stage
Cement	0	60
Hydraulic Lime	0	60

**Table 4:** Lag time between the maximum and minimum temperatures in spring climate.

Spring	Lag Time (min)	
	Cooling Stage	Heating Stage
Cement	60	0
Hydraulic Lime	60	0

**Table 5:** Lag time between the maximum and minimum temperatures in autumn climate.

Autumn	Lag Time (min)	
	Cooling Stage	Heating Stage
Cement	60	0
Hydraulic Lime	60	0

**Table 6:** Cooling and heating needs during one day.

Formulation	Cooling Needs (J/m^3^)			Heating Needs (J/m^3^)		
	Summer	Spring	Autumn	Summer	Spring	Autumn
HL_800_LS	265,855	0	0	0	254,044	255,321
HL_800_LS/PEG	265,702	0	0	0	253,572	254,947
C_800_LS	266,003	0	0	0	254,017	255,266
C_800_LS/PEG	265,230	0	0	0	253,636	255,051

**Table 7:** Energy savings per day for mortars containing PCM composite.

Formulation	Cooling Needs (J/m^3^)	Heating Needs (J/m^3^)	
	Summer	Spring	Autumn
Hydraulic Lime	152.7	472.0	374.8
Cement	772.9	381.1	214.8
